# Antimicrobial treatment for 7 versus 14 days in patients with bacteremia: a meta-analysis of randomized controlled trials

**DOI:** 10.1007/s15010-025-02562-4

**Published:** 2025-06-08

**Authors:** Marlene Prager, Felix Bergmann, Lena Pracher, Dragan Copic, Jasmin Zessner-Spitzenberg, Georg Gelbenegger, Heimo Lagler, Nicole Harrison, Heinz Burgmann, Markus Zeitlinger, Anselm Jorda

**Affiliations:** 1 Department of Clinical Pharmacology, Medical University of Vienna, Waehringer Guertel 18-20, Vienna, 1090 Austria; 2 Department of Internal Medicine III, Division of Nephrology & Dialysis, Medical University of Vienna, Vienna, Austria; 3 Department of Internal Medicine III, Division of Gastroenterology & Hepatology, Medical University of Vienna, Vienna, Austria; 4 Department of Medicine I, Division of Infectious Diseases and Tropical Medicine, Medical University of Vienna, Vienna, Austria

**Keywords:** Antibiotic, Bacteria, Gram-positive, Treatment duration, Bloodstream infection (BSI)

## Abstract

**Purpose:**

The optimal duration of antibiotic treatment in patients with bacteremia is a matter of ongoing debate.

**Methods:**

We conducted a meta-analysis of randomized controlled trials that compared 7 days with 14 days of antimicrobial treatment in adults with bacteremia. The systematic search included trials published until December 2024. Efficacy outcomes included 90-day all-cause mortality, recurrence of bacteremia and mean length of hospital stay. Safety outcomes included the total number of adverse events, *Clostridioides difficile* infections, diarrhea, acute kidney injury, rash or emergence of antibiotic resistance.

**Results:**

The final analysis included four randomized controlled trials with a total of 4790 participants. Death by day 90 occurred in 321 (13.3%) of 2406 patients receiving antibiotic treatment for 7 days and 342 (14.3%) of 2384 patients receiving antibiotic treatment for 14 days (RR 0.93 [95% CI, 0.81 to 1.07)]; *p* = 0.30; prediction interval 0.74 to 1.17). The mean hospital stay did not differ significantly (mean difference − 0.18 days [95% CI, -1.03 to 0.67]; *p* = 0.69; prediction interval − 2.57 to 2.22). Recurrence of bacteremia was similar between antibiotic treatment for 7 days (64 [2.7%] of 2406) and antibiotic treatment for 14 days (56 [2.3%] of 2384) (RR 1.14 [95% CI, 0.80 to 1.63)]; *p* = 0.47; prediction interval 0.64 to 2.03). Safety outcomes, including the total number of adverse events, *Clostridioides difficile* infections, diarrhea, acute kidney injury, rash, and antibiotic resistance, were similar between groups.

**Conclusions:**

This meta-analysis suggests that 7-day and 14-day antimicrobial treatment is associated with a similar efficacy and safety profile in patients with bacteremia.

**Supplementary Information:**

The online version contains supplementary material available at 10.1007/s15010-025-02562-4.

## Introduction

Bacteremia is a major contributor to morbidity and mortality across diverse patient populations worldwide, requiring effective antibiotic therapy [1, 2]. There are an estimated 1.8 million episodes of bloodstream infections (BSI) causing approximately 250,000 deaths annually across Europe and North America [1]. The terms BSI and bacteremia are often used interchangeably. However, while BSI can be caused by different pathogens, such as bacteria or fungi, bacteremia refers specifically to the presence of bacteria in the blood [3]. While several observational studies have recently compared different durations of antimicrobial treatment for bacteremia, the ideal duration remains unkown [5–9]. Despite increasing evidence supporting the non-inferiority of shorter courses of antibiotic therapy [4, 6, 7, 8, 10–15], prolonged treatment durations of up to 14 days are still commonly used [16, 17] and recommended by current guidelines [18, 19].

In an era of increasing antibiotic resistance and economic pressure, shortening the duration of antibiotic treatment appears to be a reasonable approach, ensuring infections are treated only for as long as necessary to achieve optimal cure rates [20, 21, 22]. However, in clinical practice, balancing the competing risks of short-course versus long-course antibiotic treatment is challenging. Shorter treatment durations raise concerns about the potential for treatment failure or recurrence of bacteremia, whereas prolonged antibiotic courses supposedly increase the risk of adverse events, including *Clostridioides difficile* (*C. difficile*) infections, promote resistance in non-targeted bacteria, and lead to avoidable healthcare costs [23, 24]. Moreover, the reduction in the duration of antibiotic therapy is considered a key component of antibiotic stewardship programs [25].

Building on recent randomized controlled trials (RCTs) [11–13] and the addition of the Bacteremia Antibiotic Length Actually Needed for Clinical Effectiveness (BALANCE) trial by Daneman et al. [10], which included over 3,600 patients, we conducted a meta-analysis evaluating the efficacy and safety of 7-day versus 14-day antimicrobial treatment in patients with bacteremia. Our goal was to offer a thorough analysis of the existing evidence to inform clinical practice and guide the development of future treatment guidelines.

## Methods

### Study registration

The present study was conducted according to the Cochrane Handbook [26] and reporting followed the Preferred Reporting Items for Systematic Reviews and Meta-Analyses (PRISMA) [27]. The study was registered at PROSPERO with the identifier CRD42024621702. Ethics approval was not required for this analysis.

### Data sources and study selection

We performed a systematic literature search and meta-analysis of randomized controlled trials to evaluate the summary effect estimate of 7-day versus 14-day antimicrobial treatment in adult patients (aged 18 years or older) with bacteremia on the 90-day all-cause mortality. The search was conducted on major online databases, including PubMed, Embase, Web of Science and the Cochrane Library. We searched for peer-reviewed trials published in English up to December 2, 2024. The primary search terms were: (bloodstream infection OR bacteremia) AND (antibiotic duration OR 7 days OR 14 days OR short-course treatment OR long-course treatment) AND (mortality OR survival OR death OR cure) as well as related Medical Subject Headings (MeSH) terms and synonyms. The complete search input per database is summarized in Supplementary Table S1. Furthermore, we screened prior meta-analyses and systematic reviews, along with references of relevant literature.

The literature search was performed independently by two authors (L.P. and M.P.), based on titles, abstracts and full text manuscripts to identify suitable trials. Discrepancies between the literature search results were solved by a third author (A.J.). The inclusion criteria were: (i) randomized controlled clinical trials, (ii) study population consisted of patients with confirmed bacteremia, (iii) comparison of 7-day versus 14-day antimicrobial treatment, (iv) trials that only included adult patients 18 years or older, (v) trials that reported 90-day all-cause mortality. We excluded non-randomized trials, retrospective studies, secondary analyses, case-reports, abstracts, and reviews.

### Data extraction and outcomes

This meta-analysis analyzed all data based on the intention-to-treat outcomes of the included studies, without the use of imputation.

The primary outcome of this analysis was the 90-day all-cause mortality during the observational period of the respective studies. Secondary outcomes included the length of hospital stay and recurrence of bacteremia.

Safety outcomes included total number of adverse events, infection with *C. difficile*, diarrhea, acute kidney injury, rash and emergence of resistance. Subgroup analyses for the primary outcome considered urinary tract infection (UTI) versus non-UTI as primary infection, detection of Gram-positive versus Gram-negative pathogen, patient age, and use of vasopressors. The subgroup ‘elderly/frail’ was defined as an age of 65 years or older in the trials by Yahav et al, von Dach et al., and Molina et al. and a Clinical Frailty Scale score of 5 or more in the trial by Daneman et al. The subgroup ‘Hypotension/Vasopressor use’ was defined as arterial systolic blood pressure below 100 mm Hg in the studies by Yahav et al., von Dach et al. and Molina et al. and as the use of vasopressors or inotropes in the study by Daneman et al.

The overall certainty of evidence for the primary outcome was assessed following the Grading of Recommendations, Assessment, Development, and Evaluations (GRADE) guidelines [28]. The risk of bias (RoB) in the included trials was assessed using the Cochrane Risk of Bias tool.

### Data synthesis and statistical analysis

We presented categorical variables as numbers with percentages (%) and summarized continuous variables as means with standard deviations (SD) or medians with interquartile ranges (IQR), depending on the data distribution. We calculated pooled risk ratios (RRs) for binary outcomes or mean differences (MDs) for continuous variables using the inverse-variance method and a random-effects model to address the variability between studies. RRs and MDs are presented with 95% confidence intervals (95% CI) and p-values for the summary estimates. We assessed heterogeneity across studies using Chi-square statistics and the Higgins I² statistic. I² values were categorized as minimal (0–25%), moderate (26–50%), high (51–75%), and very high (76–100%) inconsistency [29]. In addition to the I² statistic, we also calculated 95% prediction intervals for the primary and secondary outcomes using a random-effects model. Prediction intervals provide an estimate of the range in which the true effect sizes of future similar studies are expected to lie [30, 31]. Our subgroup analysis utilized tests for heterogeneity to investigate differences between subgroups. Sensitivity analyses were performed by switching the analysis to a fixed-effects framework, in which the Mantel-Haenszel method was utilized, and by sequentially omitting each individual trial. Median with IQRs were converted to means with SDs using methods suggested by the Cochrane Handbook.

We reported unadjusted p-values. P-values below a value of 0.05 were considered statistically significant. The statistical analyses were performed using Review Manager (version 5.4.1, 2014, Copenhagen: The Nordic Cochrane Centre, The Cochrane Collaboration) and R (R version 2024.04.2 + 764, Vienna, Austria).

## Results

**Fig. 1:**
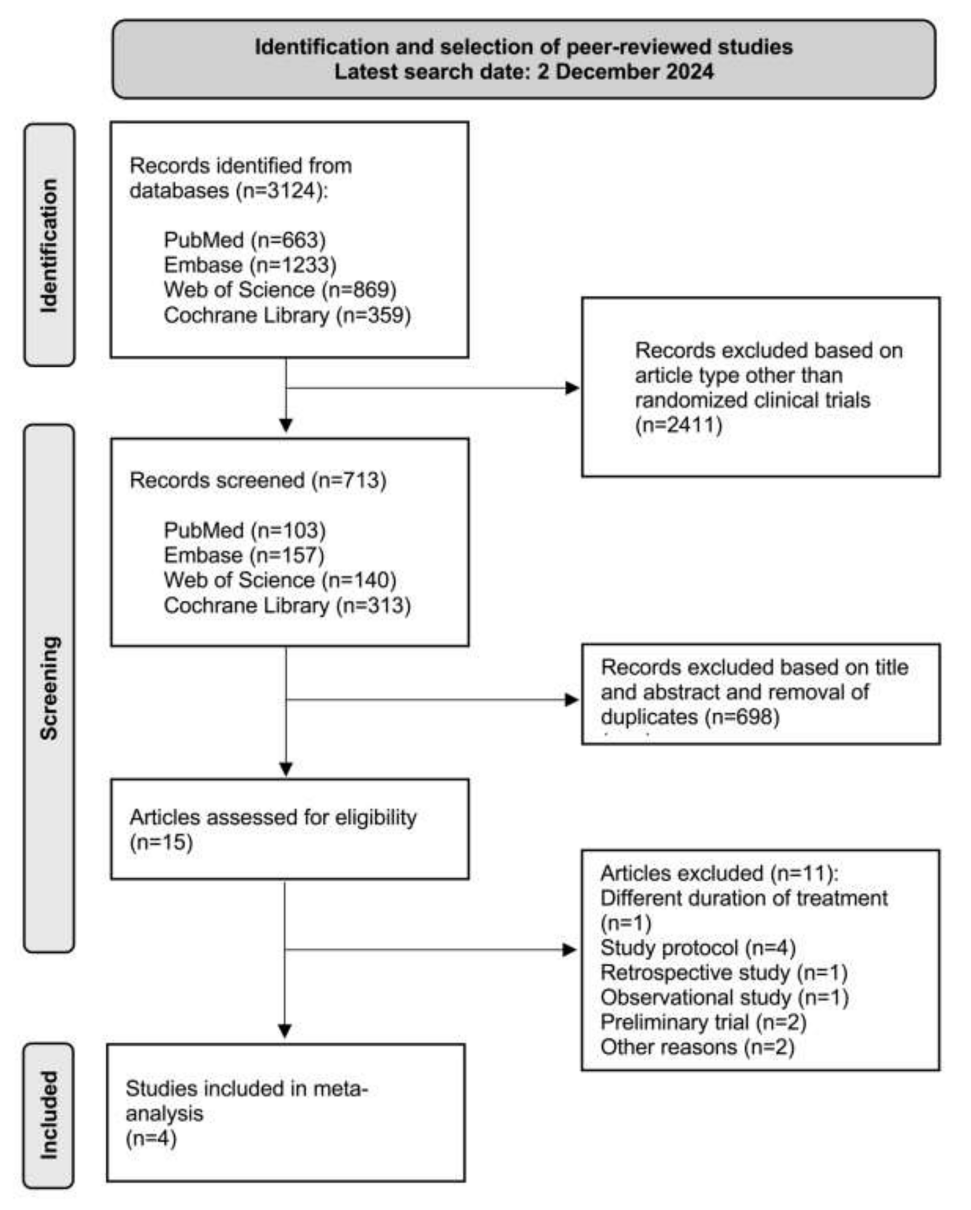
PRISMA flow chart of study identification and selection

A total of 3124 publications were identified through the initial literature search on December 2, 2024. After the exclusion of 3120 articles not meeting the pre-specified criteria, a total of four articles remained. Figure 1 depicts the flow chart of the selection process.

### Study characteristics

Our final analysis included four randomized controlled trials with a total of 4790 participants [10–13]. Key characteristics of the included trials are presented in Table 1. Table S2 provides extended study characteristics. The study by Yahav et al. had 3 study sites in Israel and Italy, the study of von Dach et al. had 3 study sites in Switzerland, and the study by Molina et al. had 5 study sites in Spain. The trial by Daneman et al. was the largest with 74 sites across Canada, United States, Australia, New Zealand, Saudi Arabia, Israel and Switzerland. The sample sizes ranged from 248 (Molina et al.) to 3608 (Daneman et al.). In the pooled analysis, 1161 (48.2%) of 2408 participants in the 7-day group and 1162 (48.7%) of 2386 participants in the 14-day group were female (Supplementary Table S3). Common comorbidities were diabetes (7-day group vs. 14-day group: 32.1% vs. 30.0%), solid-organ cancer (20.6% vs. 19.8%), obesity (18.2% vs. 18.1%), and renal insufficiency (20.8% vs. 21.1%). The majority of infections were community-acquired (73.2% vs. 71.2%). The most common source of bacteremia was the urinary tract (47.6% vs. 48.1%), followed by intraabdominal (17.7% vs. 17.3%), pulmonary (10.8% vs. 11.7%) and catheter-associated infections (6.2% vs. 6.6%). *Escherichia coli* was the most common causative pathogen (49.4% vs. 45.0%), followed by Klebsiella species (15.7% vs. 15.1%).

**Table 1:** Key study characteristics of the included trials

Study	Year	Geographical Region	Number of Sites	Study Population	Total Sample Size	Primary Outcome	Follow-Up Period ^b^
Yahav et al.	2019	Israel, Italy	3	Hospitalized with uncomplicated Gram-negative bacteremia	604	Composite of all-cause mortality, recurrence of bacteremia, complications, or extended hospitalization	90 days
von Dach et al.	2020	Switzerland	3	Hospitalized with uncomplicated Gram-negative bacteremia	334 ^a^	Composite of all-cause mortality, recurrence of bacteremia, complications, or restart of therapy	90 days
Molina et al.	2022	Spain	5	Hospitalized with Enterobacterales bacteremia	248	Number of days of antibiotic treatment	28 days
Daneman et al.	2024	Canada, Australia, New Zealand, United States, Saudi Arabia, Israel, Switzerland	74	Hospitalized with bacteremia	3608	All-cause mortality	90 days

^a^ The C-reactive protein (CRP) based duration arm was excluded from the analysis, therefore leaving a total sample size of 334

^b^ Refers to the follow up period of the respective study’s primary outcome

### Efficacy outcomes

**Fig. 2:**
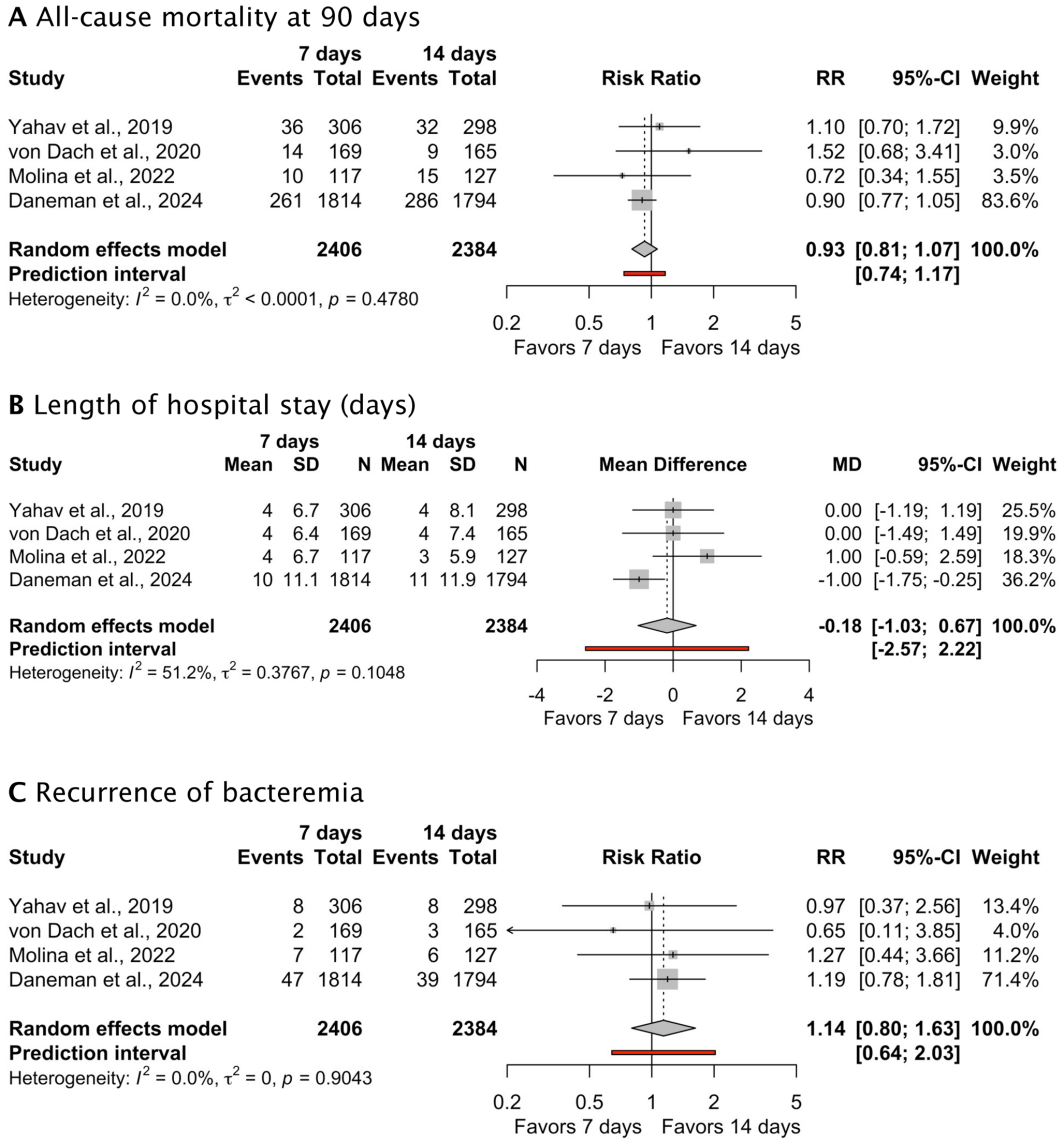
Forrest plot depicting the efficacy outcomes of 7 versus 14 days of antimicrobial treatment (**A**) All-cause mortality at 90 days (**B**) Length of hospital stay (days) (**C**) Recurrence of bacteremia

The efficacy and safety outcomes are summarized in Table 2. The all-cause mortality at 90 days was similar between the 7-day and 14-day treatment groups. Death by day 90 occurred in 321 (13.3%) of 2406 patients receiving antibiotic treatment for 7 days and 342 (14.3%) of 2384 patients receiving antibiotic treatment for 14 days (RR 0.93 [95% CI, 0.81 to 1.07)]; *p* = 0.30; I^2^ = 0%; prediction interval 0.74 to 1.17) (Fig. 2A).

**Table 2:** Efficacy and safety outcomes of the included trials

	Studies	7-Day Group	14-Day Group	Risk difference (95% CI)	*P* value of overall effect	I^2^ (%)
**Efficacy outcomes**						
90-day all-cause mortality, n/N (%)	^10–13^	321/2406 (13.3)	342/2384 (14.3)	0.93 (0.81–1.07)	0.30	0
Length of hospital stay (days) mean ± SD	^10–13^	8.52 ± 10.18	9.21 ± 10.97	-0.18 (-1.04–0.68)	0.69	51
Recurrence of bacteremia, n/N (%)	^10–13^	64/2406 (2.7)	56/2384 (2.3)	1.14 (0.80–1.63)	0.47	0
**Safety outcomes**						
Number of adverse events, n/N (%)	^11–13^	160/592 (27.0)	178/590 (30.2)	0.91 (0.77–1.08)	0.30	0
*C. difficile* infection, n/N (%)	^10,11,13^	36/2289 (1.6)	40/2257 (1.8)	0.88 (0.56–1.38)	0.58	0
Diarrhea, n/N (%)	^11–13^	53/592 (9.0)	58/590 (9.8)	0.89 (0.63–1.25)	0.51	0
Acute kidney injury, n/N (%)	^10–12^	32/2433 (1.3)	31/2384 (1.3)	1.01 (0.62–1.65)	0.96	0
Rash, n/N (%)	^11–13^	4/592 (0.7)	9/590 (1.5)	0.46 (0.14–1.55)	0.21	0
Emergence of resistance, n/N (%)	^10,11,13^	56/2289 (2.4)	53/2257 (2.3)	1.02 (0.68–1.52)	0.93	10

The mean length of hospital stay did not differ between patients receiving antibiotic treatment for 7 days or for 14 days (Mean difference − 0.18 days [95% CI, -1.03 to 0.67)]; *p* = 0.69; I^2^ = 51%; prediction interval − 2.57 to 2.22) (Fig. 2B).

Recurrence of bacteremia was similar between antibiotic treatment for 7 days (64 [2.7%] of 2406) and antibiotic treatment for 14 days (56 [2.3%] of 2384) (RR 1.14 [95% CI, 0.80 to 1.63)]; *p* = 0.47; I^2^ = 0%; prediction interval 0.64 to 2.03) (Fig. 2C).

### Safety outcomes

**Fig. 3:**
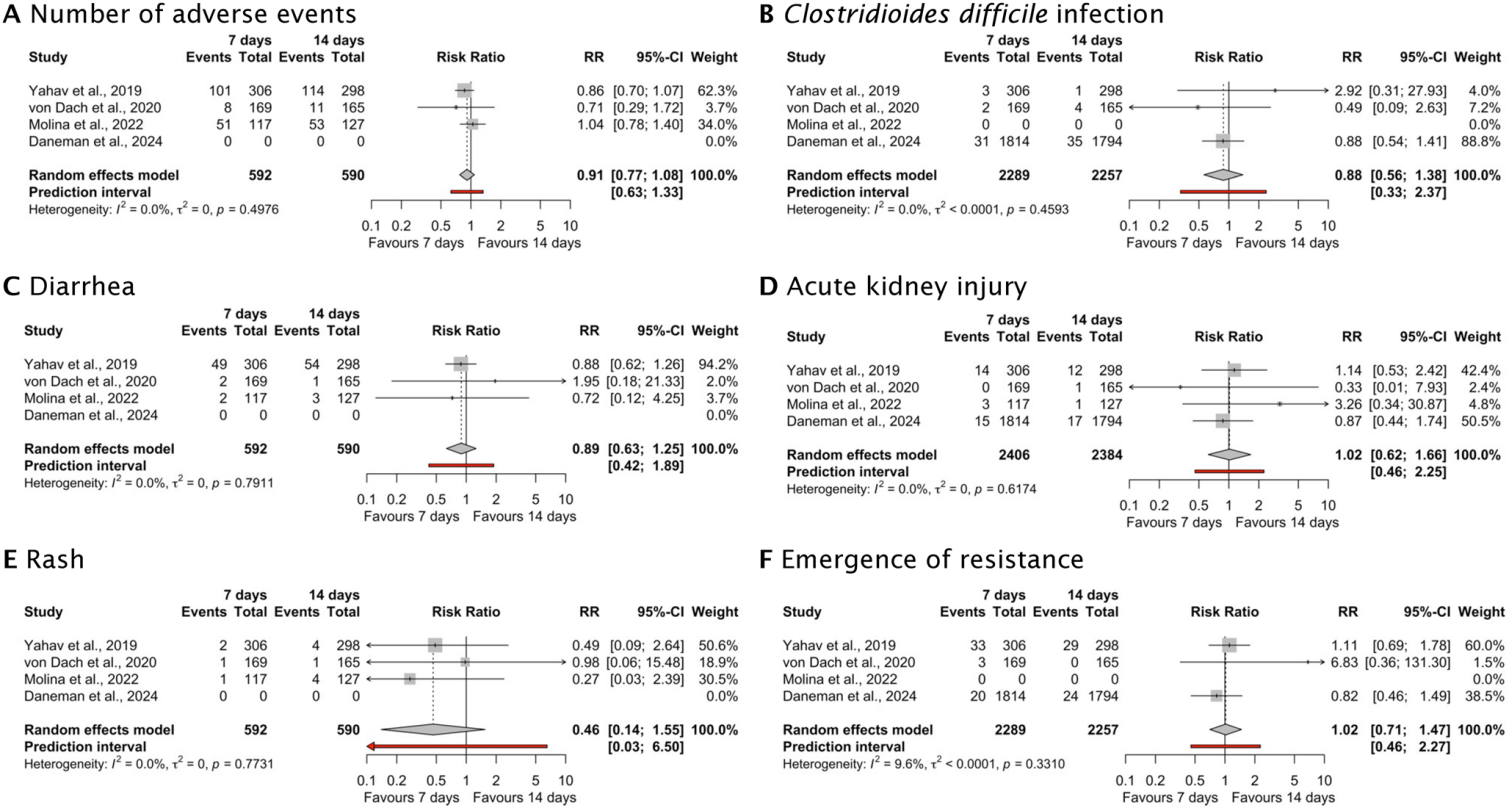
Forrest plot depicting the safety outcomes of 7 versus 14 days of antimicrobial treatment (**A**) Number of adverse events, (**B**) *C. difficile* infection, (**C**) Diarrhea, (**D**) Acute kidney injury, (**E**) Rash, (**F**) Emergence of resistance

The number of participants experiencing any adverse event was similar between the 7-day group (160 [27.0%] of 592) and 14-day group (178 [30.2%] of 590), and the rates were associated with a similar relative risk (RR 0.91 [95% CI, 0.77 to 1.08)]; *p* = 0.30; I^2^ = 0%; prediction interval 0.63 to 1.33) (Fig. 3A). Infection with *C. difficile* occurred in 36 (1.6%) of 2289 patients receiving antibiotic treatment of 7 days and 40 (1.8%) of 2257 patients receiving antibiotic treatment for 14 days (RR 0.88 [95% CI, 0.56 to 1.38)]; *p* = 0.58; I^2^ = 0%; prediction interval 0.33 to 2.37) (Fig. 3B). Diarrhea occurred in 53 (9.0%) of 592 patients receiving antibiotic treatment for 7 days and 58 (9.8%) of 590 patients receiving antibiotic treatment for 14 days (RR 0.89 [95% CI, 0.63 to 1.25)]; *p* = 0.51; I^2^ = 0%; prediction interval 0.42 to 1.89) (Fig. 3C).

Acute kidney injury occurred in 32 (1.3%) of 2433 patients receiving antibiotic treatment of 7 days and 31 (1.3%) of 2384 patients receiving antibiotic treatment for 14 days (RR 1.02 [95% CI, 0.62 to 1.66)]; *p* = 0.96; I^2^ = 0%; prediction interval 0.46 to 2.25) (Fig. 3D). A rash was observed in 4 (0.7%) of 592 patients in the 7-day group and 9 (1.5%) of 590 patients in the 14-day group (RR 0.46 [95% CI, 0.14 to 1.55)]; *p* = 0.21; I^2^ = 0%; prediction interval 0.03 to 6.5) (Fig. 3E). The emergence of resistance was documented in 56 (2.4%) of 2289 patients in the 7-day group and 53 (2.3%) of 2257 patients in the 14-day group (RR 1.02 [95% CI, 0.71 to 1.47)]; *p* = 0.93; I^2^ = 10%; prediction interval 0.46 to 2.27) (Fig. 3F).

### Subgroup analyses

**Fig. 4:**
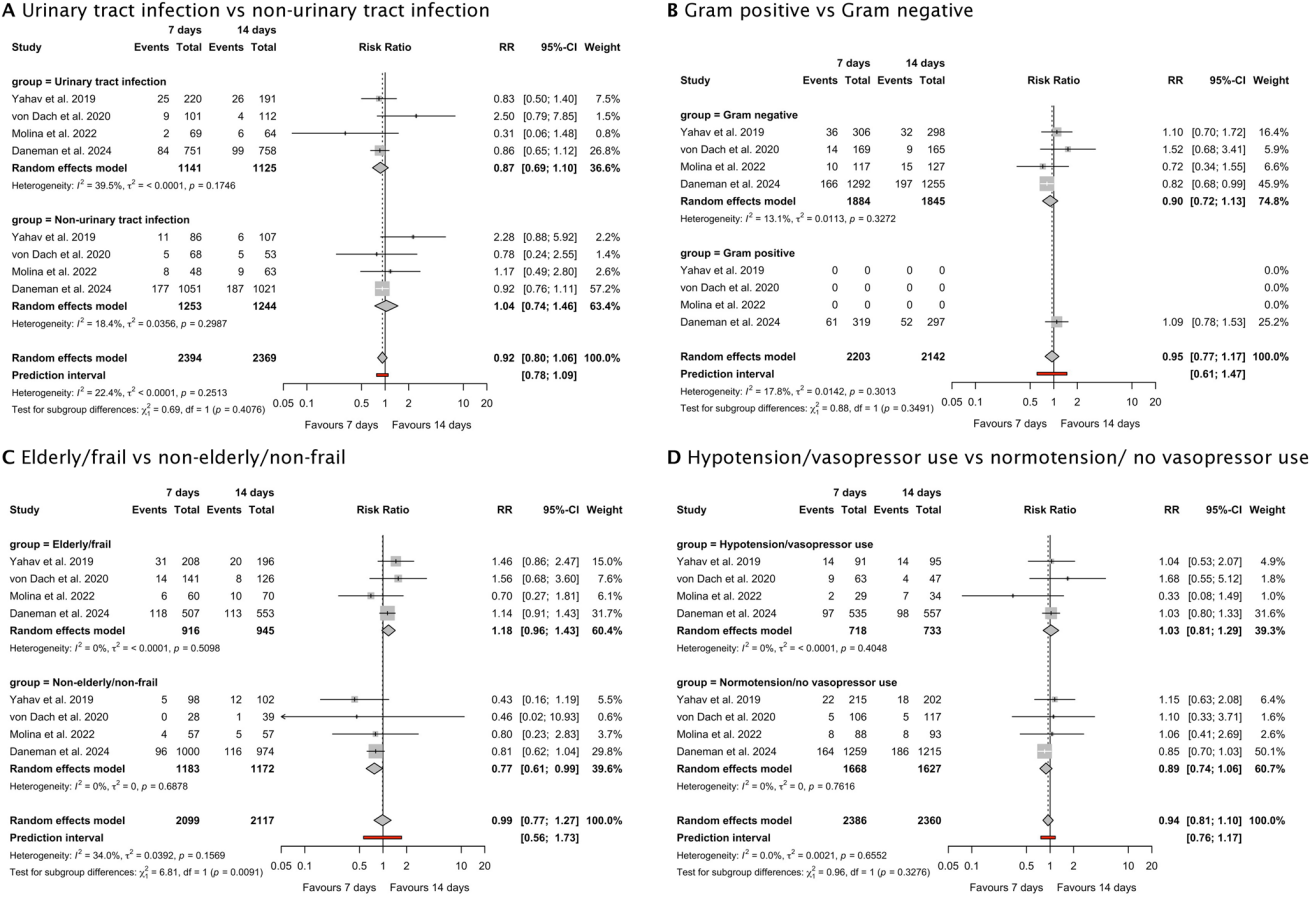
Forrest plot depicting the subgroup analyses of the 90-day all-cause mortality of 7- versus 14-days of antimicrobial treatment (**A**) Urinary tract infection vs. non-urinary tract infection, (**B**) Gram-positive vs. Gram-negative, (**C**) Elderly/frail vs. non-elderly/non-frail, (**D**) Hypotension/vasopressor use vs. normotension/no vasopressor use. *Note:* The subgroup ‘Elderly/Frail’ was defined as an age of 65 years or older in the trials by Yahav et al., von Dach et al., and Molina et al. and a Clinical Frailty Scale score of 5 or more in the trial by Daneman et al. The subgroup ‘Hypotension/Vasopressor use’ was defined as arterial systolic blood pressure below 100 mm Hg in the studies by Yahav et al., von Dach et al. and Molina et al. and as the use of vasopressors or inotropes in the study by Daneman et al.

No treatment effect heterogeneity regarding all-cause mortality at 90 days was observed in the comparison between antibiotic treatment for 7 days versus 14 days in the subgroup of patients with urinary tract infections (RR 0.87 [95% CI, 0.69 to 1.10]; *p* = 0.59) and patients with other primary sites of infection (RR 1.04 [95% CI, 0.74 to 1.46]; *p* = 0.87) (Chi^2^ = 0.69, *p* = 0.41, I^2^ = 22.4%) (Fig. 4A).

No treatment effect heterogeneity regarding all-cause mortality at 90 days was observed in the comparison between antibiotic treatment for 7 days versus 14 days in the subgroup of patients with Gram-negative bacteremia (RR 0.90 [95% CI, 0.72 to 1.13]; *p* = 0.31) and patients with Gram-positive bacteremia (RR 1.09 [95% CI, 0.78 to 1.53]; *p* = 0.87) (Chi^2^ = 0.88, *p* = 0.35, I^2^ = 18%) (Fig. 4B).

A significant treatment effect heterogeneity regarding all-cause mortality at 90 days was observed between the subgroups of elderly/frail patients versus non-elderly/non-frail patients (Chi^2^ = 6.81, *p* = 0.009, I^2^ = 34%) (Fig. 4C). In the subgroup of elderly or frail patients, death by day 90 occurred in 169 (18.5%) of 916 patients in the 7-day group and 151 (16.0%) of 945 patients in the 14-day group (RR 1.18 [95% CI, 0.96 to 1.43)]; *p* = 0.11). In the subgroup of non-elderly or non-frail patients, antibiotic treatment for 7 days (105 [8.9%] of 1183) was associated with a significantly reduced all-cause mortality at 90 days compared to antibiotic treatment for 14 days (134 [11.4%] of 1172) (RR 0.77 [95% CI, 0.61 to 0.99)]; *p* = 0.04).

No treatment effect heterogeneity regarding all-cause mortality at 90 days was observed in the comparison between antibiotic treatment for 7 days versus 14 days in the subgroup of patients with hypotension or vasopressor use (RR 1.03 [95% CI, 0.81 to 1.29]; *p* = 0.83) and patients with normotension and without vasopressor use (RR 0.89 [95% CI, 0.74 to 1.06]; *p* = 0.19) (Chi^2^ = 0.96, *p* = 0.33, I^2^ = 0%) (Fig. 4D).

### Sensitivity analyses

The sequential omission of each individual trial had no significant effect on any of the efficacy and safety outcomes. Changing the random-effects model to a fixed-effects model also had no significant effect on any of the efficacy and safety outcomes.

### Risk of bias and certainty of evidence

All included studies were considered to have some risk of bias. The primary risk of bias throughout all trials was related to deviations from the intended interventions due to the unblinded study design or some patients requiring prolonged or resumed treatments, particularly in the 7-day group. As can be seen in Table S5, we rated the certainty of evidence as low, mainly due to the potential risk of bias and high imprecision, which arises due to the relatively wide confidence intervals of the primary outcome.

## Discussion

The present study included four large RCTs and incorporated data from a total of 4790 patients. By including the BALANCE trial by Daneman et al., more than 3600 patients were added to the analysis - hence further strengthening the already existing evidence [10].

In our analysis, the 7-day and 14-day antimicrobial treatment durations were associated with a similar risk of all-cause mortality, recurrence of bacteremia and mean length of hospital stay. Importantly, the 7-day treatment regimen did not lead to increased mortality or recurrence, aligning with prior evidence that supports its safety and efficacy as an alternative to longer antibiotic courses [15].

Also, 7-day versus 14-day antibiotic treatment revealed comparable safety outcomes with no significant differences between the number of adverse events, *C. difficile* infections, diarrhea, acute kidney injury, rash or antibiotic resistance. These findings contrast previous trials that reported trends towards an increased risk of resistance development [9], or superinfections [8, 32] when treated for longer than 10 days. Although the safety analysis demonstrated similar results in both groups, a shorter duration of antimicrobial treatment offers further benefits, such as a significant reduction in the financial burden on the healthcare system. Previous analyses have estimated over 1,200,000 episodes of BSI per year in Europe [1]. Transitioning just half of these patients to a shorter treatment could result in savings of up to €1.6 billion across Europe, as estimated by Daneman et al. [33].

Our analysis showed no treatment effect heterogeneity regarding all-cause mortality at 90 days across most subgroups, including those defined by infection site (urinary versus non-urinary tract infection), pathogen type (Gram-positive versus Gram-negative), and hemodynamic status (hypotension/vasopressor use versus normotension/no vasopressor use). However, significant heterogeneity was observed in non-elderly or non-frail patients, where a trend favored 14-day treatment, while non-elderly or non-frail patients benefited from a significantly reduced mortality with a 7-day treatment. Notably, there is also a risk that the results of these subgroup analyses are false positive.

The trials included in this meta-analysis involved patients with varying baseline health and disease severity, enhancing generalizability but also introducing greater heterogeneity. In the largest trial included, more than 50% of patients were admitted to the ICU at the time of enrollment. Thus, a significant proportion of patients included in this analysis were critically ill, suggesting that the 7-day and 14-day treatment regimens have similar safety and efficacy profiles, even in higher-risk patients.

In the trials by Yahav et al., von Dach et al., and Molina et al., the mean hospital stay was short with approximately 4 days for both treatment groups. In contrast, the trial by Daneman et al. reported slightly longer stays, with a mean of 10 days for the 7-day group and 11 days for the 14-day group [10, 11, 12, 13]. This discrepancy in hospital stay durations across trials may reflect differences in healthcare systems or discharge policies. Moreover, the higher mean Sequential Organ Failure Assessment (SOFA) score of about 5 in the trial by Daneman et al., compared to about 2 in the trial by Yahav et al., and a quick SOFA (qSOFA) score of about 1 in the trial by von Dach et al., indicate that patients in the trial by Daneman et al. were more critically ill.

We acknowledge several limitations of this meta-analysis. First, trial-level meta-analyses, compared to individual-participant level analyses, have inherent limitations [34]. Second, while the choice of antibiotic, frequency and route of administration was at the discretion of the treating physician in all included trials, this reflects real-world practice but also introduces variability. However, this variability is unlikely to have significantly influenced the outcomes, as all trials ensured the use of adequate antibiotic therapy based on local guidelines or laboratory susceptibility reports [10, 11, 12, 13]. Third, in contrast to the other trials, the trial by Daneman et al. included Gram-positive in addition to Gram-negative bacteria. Despite this broader scope, most patients had monomicrobial Gram-negative bacteremia, making the findings largely representative of Gram-negative infections. This aligns with the patient populations in the other three trials but may limit the applicability of the results to specific patient populations with mixed or predominantly Gram-positive infections. Forth, the definitions of subgroups (‘elderly/frail’ and ‘vasopressor use/hypotension’) were inconsistent, but were restricted to the data published by the individual studies. Besides subgroup analyses based on the Gram staining of the causative pathogens, we were not able to perform more detailed subgroup analyses by specific bacterial species. Lastly, while it remains debated whether 30-day or 90-day mortality more accurately reflects the impact of antimicrobial therapy, most of the included trials only reported 90-day mortality, which formed the basis of our analysis. While shorter observation periods may better assess the direct impact of antimicrobial treatment, longer observation periods can better capture late complications or relapses occurring after hospital discharge.

## Conclusion

Our meta-analysis demonstrated that 7 days and 14 days of antimicrobial treatment in patients with bacteremia had similar outcomes in terms of all-cause mortality, recurrence of bacteremia, mean length of hospital stay, and safety endpoints. These findings support the use of shorter antibiotic courses as a safe and effective alternative. However, subgroup analyses suggest that specific patient populations, such as younger and less frail patients, may particularly benefit from shorter treatment regimens. To better understand optimal treatment durations, further research is needed in special populations and in patients with Gram-positive or polymicrobial infections.

## Electronic supplementary material

Below is the link to the electronic supplementary material.

Supplementary Material 1

## Data Availability

No datasets were generated or analysed during the current study.
