# Supplementary material for: Antimicrobial treatment for 7 versus 14 days in patients with bacteremia: a meta-analysis of randomized controlled trials
Source: Infection. 2025 Jun 8;53(5):2159–68. doi: 10.1007/s15010-025-02562-4 (PMC12460493; doi:10.1007/s15010-025-02562-4)
Supplement: Supplementary file 1 — Supplementary Material 1 [file 15010_2025_2562_MOESM1_ESM.pdf]

Supplementary Appendix of Meta-Analysis

Antimicrobial treatment for 7 versus 14 days in patients with bacteremia: A meta-analysis of randomized controlled trials

Marlene Prager[1], Felix Bergmann[1], Lena Pracher[1], Dragan Copic[2], Jasmin Zessner-Spitzenberg[3], Georg Gelbenegger[1], Heimo Lagler[4], Nicole Harrison[4], Heinz Burgmann[4], Markus Zeitlinger[1*], Anselm Jorda[1]

[1]Department of Clinical Pharmacology, Medical University of Vienna, Vienna, Austria
[2]Department of Internal Medicine III, Division of Nephrology & Dialysis, Medical University of Vienna, Vienna, Austria
[3]Department of Internal Medicine III, Division of Gastroenterology & Hepatology, Medical University of Vienna, Vienna, Austria
[4]Department of Medicine I, Division of Infectious Diseases and Tropical Medicine, Medical University of Vienna, Vienna, Austria

Table S1 Precise search strategy and number of records found in each database (Search conducted on December 2, 2024)

	N
**PubMed**	
("bloodstream infection"[Title/Abstract] OR "bacteremia"[Title/Abstract]) AND ("antibiotic duration"[Title/Abstract] OR "7 days"[Title/Abstract] OR "14 days"[Title/Abstract] OR "short-course treatment"[Title/Abstract] OR "long-course treatment"[Title/Abstract]) AND ("mortality"[Title/Abstract] OR "survival"[Title/Abstract] OR "death"[Title/Abstract] OR "cure"[Title/Abstract])	**663**
("bloodstream infection"[Title/Abstract] OR "bacteremia"[Title/Abstract]) AND ("antibiotic duration"[Title/Abstract] OR "7 days"[Title/Abstract] OR "14 days"[Title/Abstract] OR "short-course treatment"[Title/Abstract] OR "long-course treatment"[Title/Abstract]) AND ("mortality"[Title/Abstract] OR "survival"[Title/Abstract] OR "death"[Title/Abstract] OR "cure"[Title/Abstract]) AND ("randomized"[Title/Abstract] OR "randomised"[Title/Abstract] OR "randomly"[Title/Abstract])	**103**
**Embase**	
('bloodstream infection':ti,ab OR 'bacteremia':ti,ab) AND ('antibiotic duration':ti,ab OR '7 days':ti,ab OR '14 days':ti,ab OR 'short-course treatment':ti,ab OR 'long-course treatment':ti,ab) AND ('mortality':ti,ab OR 'survival':ti,ab OR 'death':ti,ab OR 'cure':ti,ab)	**1233**
('bloodstream infection':ti,ab OR 'bacteremia':ti,ab) AND ('antibiotic duration':ti,ab OR '7 days':ti,ab OR '14 days':ti,ab OR 'short-course treatment':ti,ab OR 'long-course treatment':ti,ab) AND ('mortality':ti,ab OR 'survival':ti,ab OR 'death':ti,ab OR 'cure':ti,ab) AND ('randomized':ti,ab OR 'randomised':ti,ab OR 'randomly':ti,ab)	**157**
**Web of Science**	
TS=("bloodstream infection" OR "bacteremia") AND TS=("antibiotic duration" OR "7 days" OR "14 days" OR "short-course treatment" OR "long-course treatment") AND TS=("mortality" OR "survival" OR "death" OR "cure")	**869**
TS=("bloodstream infection" OR "bacteremia") AND TS=("antibiotic duration" OR "7 days" OR "14 days" OR "short-course treatment" OR "long-course treatment") AND TS=("mortality" OR "survival" OR "death" OR "cure") AND TS=("randomized" OR "randomised" OR "randomly")	**140**
**Cochrane Library**	
(("bloodstream infection" OR "bacteremia") AND ("antibiotic duration" OR "7 days" OR "14 days" OR "short-course treatment" OR "long-course treatment") AND ("mortality" OR "survival" OR "death" OR "cure")):ti,ab,kw	**359**
(("bloodstream infection" OR "bacteremia") AND ("antibiotic duration" OR "7 days" OR "14 days" OR "short-course treatment" OR "long-course treatment") AND ("mortality" OR "survival" OR "death" OR "cure") AND ("randomized" OR "randomised" OR "randomly")):ti,ab,kw	**313**

Table S2 Extended trial characteristics

Trial	Region	Study Population	Recruit-ment Dates	Blinding	Random ized	No. of Sites	ITT Sample Size 7-Day Group	ITT Sample Size 14-Day Group	Primary Outcomes	Secondary Outcomes	Main Safety Outcomes	Subgroup Analyses	Key Inclusion Criteria	Key Exclusion Criteria	Max Follow -Up
**Yahav et al., 2019**	Israel, Italy	Hospitalized adult patients with aerobic gram-negative bacteremia on day 7 of appropriate antibiotic therapy (hemo-dynamically stable afebrile for at least 48 hours).	January 2013 - August 2017	open-label	yes	3	306	298	Composite of all-cause mortality, clinical failure (relapse, local suppurative complications, or distant complications), and readmission or extended hospital stay (>14 days) at 90 days from randomization.	Development of new documented infection by 90 days; functional capacity at day 30 and time to return to baseline activity by day 90; total hospital days among survivors and among all patients by 90 days; total antibiotic days by 90 days and duration of appropriate antibiotic treatment; development of resistance, defined as secondary clinical isolates resistant to 1 or more of the antibiotics used to treat the index gram-negative bacteremia; adverse events, including *C. difficile* infection.	Acute kidney injury, Liver function abnormalities, Diarrhea during hospital stay, Diarrhea until day 90, Rash, *C. difficile* infection.	The primary outcome analyses included prespecified subgroups based on patients receiving either covering (appropriate) or noncovering (inappropriate) empirical antibiotics, the source of bacteremia (urinary tract infection or another source), and whether the gram-negative bacteremia was caused by multidrug-resistant (MDR) or non-MDR gram-negative bacteria.	1. Adults ≥ 18 years old<br>2. Gram-negative bacteremia during hospital stay<br>3. Receiving appropriate antibiotic treatment for ≤7 days, hemo-dynamically stable, afebrile for the last 48 h<br>4. Source of bacteremia:<br>a. Primary bacteremia/ unknown source<br>b. Urinary tract<br>c. Abdominal<br>d. Respiratory tract<br>e. explanted Central venous catheter<br>f. Skin and soft tissue<br>4. Either community or hospital acquired bacteremia.	1. Source of bacteremia:<br>a. Endocarditis/ endovascular infections<br>b. Necrotizing fasciitis<br>c. Osteomyelitis<br>d. Abdominal abscesses<br>e. Central nervous system infections<br>f. Empyema<br>g. Central venous catheter (CVC)-related or CVC-associated bloodstream infections when the catheter is retained.<br>2. Polymicrobial growth in blood cultures involving Gram-positive or anaerobes<br>3. Specific pathogens including:<br>a. Salmonella spp.<br>b. Brucella spp.<br>4. HIV infection, Allogeneic hematopoietic stem-cell transplantation, Neutropenia.<br>6. Repeated positive blood cultures of the same organism separated by at least 24 h, regardless of antibiotic treatment<br>7. Uncontrolled focus of infection<br>8. Fever > 38C.	90 days
**von Dach et al., 2020**	Switzerland	Adults aged 18 years or older were eligible for randomization on day 5 (±1 day) of effective treatment for fermenting gram-negative bacteria in blood cultures, (afebrile for 24 hours and no signs of complicated infection (e.g., abscess) or severe immunosuppressi on).	April 2017 - May 2019	double-blind	yes	3	169	165	Clinical failure rate at day 30, defined as the presence of at least one of the following: recurrent bacteremia, local suppurative complication, distant complication, restarting gram-negative-directed antibiotic therapy due to clinical worsening, or death due to any cause.	Clinical failure rate on day 90 of follow-up.	*C difficile* infection, Diarrhea, Rash, Pruritis, Glossitis ,Thrush, Tongue discoloration, Abdominal pain, Catheter iinfection, Elevated creatinine, Headache, Fever, QT prolongation.	none	Growth of gram-negative bacteria in at least one blood culture and the administration of an effective antibiotic.	Hemodynamic instability in the 24 hours prior to recruitment, severe immunosuppression, bacteremia with nonfermenting bacilli or polymicrobial, gram-positive growth, recurrent bacteremia, or complicated infections (e.g., abscess, endocarditis).	90 days
**Molina et al., 2022**	Spain	Adults aged 18 years with bloodstream infections caused by members of the Enterobacterales.	Septemb er 2014 - Septemb er 2016	open-label	yes	5	119	129	Total number of days of antibiotic treatment prescribed to the patient for any reason, from the day of the first positive blood sample collection until the end of the follow-up.	Relapse of the eBSI, relapse of fever, clinical cure, crude mortality, superinfections, and adverse events at the end of follow-up.	Blood and lymphatic, Cardiac disorders, Gastrointestinal disorders, General disorders and administration site conditions, Hepatobiliary disorders, Infections and infestations, Injury, poisoning and procedural complications, Metabolism and nutrition disorders, Musculoskeletal and connective tissue disorders, Neoplasms, Nervous system disorders, Psychiatric disorders, Renal and urinary disorders, Respiratory, thoracic and mediastinal disorders, Skin, Vascular disorders.	none	Age > 18, hospitalized or outpatient, with Enterobacterial bloodstream infection.	(a) pregnancy, (b) blood stream infection with a noncontrolled source and no expectation of being controlled in the subsequent 24 h, (c) patients undergoing chemotherapy with neutropenia <500 cells/mm^3 expected for more than 7 days,<br>(d) eBSI secondary to infections requiring prolonged antibiotic treatment (e.g. osteomyelitis, meningitis, prostatitis, etc.), (e) concomitant infection requiring antibiotic treatment at the time of the diagnosis of the blood stream infection,<br>(f) blood stream infection caused by a carba-penemase producing member of the Enterobacterales (g) polymicrobial bacteremia, (h) expectation of survival <48 h.	28 days
**Daneman et al., 2024**	Canada, Australia, New Zealand, United States, Saudi Arabia, Israel, Switzerland	Hospitalized adult patients with bloodstream infection.	October 2014 - May 2023	open-label	yes	74	1814	1794	Death from any cause by 90 days after diagnosis of the bloodstream infection.	Death in the hospital, death in the ICU among the patients enrolled in the ICU or admitted to the ICU after the diagnosis of a bloodstream infection, relapse of bacteremia, allergy to the antibiotic and adverse events, *C. difficile* infection in the hospital, secondary infection or colonization with antimicrobial-resistant organisms, length of stay in the ICU and number of ICU-free days, length of stay in the hospital and number of hospital-free days, duration of invasive mechanical ventilation and number of ventilation-free days, number of antibiotic-free days, and duration of vasopressor use and number of vasopressor-free days.	Allergy, Anaphylaxis, Acute kidney injury, Acute hepatitis.	Acquisition of bacteremia (Hospital/ICU or community), Enrollment location (ICU or hospital ward), APACHE II Score (<25 or >25), Vasopressor use, Clinical Frailty Scale score (<5 or ≥5), Source of bacteremia, Pathogen (Gram-positive or Gram-negative or Polymicrobial).	Positive blood culture with pathogenic bacteria, hospitalized patient.	Severe immune system compromise, prosthetic heart valve or synthetic endovascular graft, infective endocarditis, osteomyelitis/septic arthritis, undrainable/undrained abscess, unremovable/ unremoved prosthetic associated infection, positive blood culture with a common contaminant organism, blood culture with *Staphylococcus aureus* or *Staphylococcus lugdunensis,* blood culture with Candida spp. or other fungal species.	90 days

Table S3 Baseline characteristics of the study populations in the included trials

	**Yahav et al., 2019**		**von Dach et al., 2020**		**Molina et al., 2022**		**Daneman et al., 2024**		**Pooled population**	
	7-Day Group	14-Day Group	7-Day Group	14-Day Group	7-Day Group	14-Day Group	7-Day Group	14-Day Group	7-Day Group	14-Day Group
Population size, n	306	298	169	165	119	129	1814	1794	2408	2386
Age, mean ± SD	71.3 ± 14.2	71.0 ± 21.6	77.7 ± 12.7	77.3 ± 13.5	65.3 ± 18.8	66.0 ± 18.0	69.3 ± 16.3	69.7 ± 15.6	70.9 ± 5.17	71.0 ± 4.70
Female sex, no. (%)	156 (51.0)	163 (54.7)	107 (63.0)	94 (57.0)	58 (49.2)	59 (45.7)	840 (46.3)	846 (47.2)	1161/2408 (48.2)	1162/2386 (48.7)
BMI, mean ± SD	N.A.	N.A.	26.3 ± 5.23	26.0 ± 4.49	N.A.	N.A.	N.A.	N.A.	26.3 ± 5.23	26.0 ± 4.49
Mean SOFA score on day 0, mean ± SD [a]	2 ± 1.5	2 ± 1.5	N.A.	N.A.	N.A.	N.A.	4.67 ± 4.45	5.0 ± 4.45	3.34 ± 1.89	3.5 ± 2.12
qSOFA score, mean ± SD [b]	N.A.	N.A.	1 ± 1.5	0.67 ± 0.75	N.A.	N.A.	N.A.	N.A.	1 ± 1.5	0.67 ± 0.75
Enrolled in ICU, no. (%)	N.A.	N.A.	N.A.	N.A.	N.A.	N.A.	997 (55.0)	989 (55.1)	997/1814 (55.0)	989/1794 (55.1)
Enrolled in hospital ward, no. (%)	N.A.	N.A.	N.A.	N.A.	N.A.	N.A.	817 (45.0)	805 (44.9)	817/2408 (45.0)	805/2386 (44.9)
Receiving mechanical ventilation, no. (%)	N.A.	N.A.	N.A.	N.A.	N.A.	N.A.	374 (20.6)	392 (21.9)	374/2408 (20.6)	392/1794 (21.9)
Charlson Comorbidity Index score, mean ± SD [c]	2 ± 1.5	2.3 ± 2.2	1 ± 1.5	1 ± 1.5	N.A.	N.A.	N.A.	N.A.	1.5 ± 0.71	1.65 ± 0.92
Charlson Score ≥ 3; no (%)	N.A.	N.A.	N.A.	N.A.	54/119 (45.4)	56/129 (43.4)	N.A.	N.A.	54/119 (45.4)	56/129 (43.4)
**Co-existing conditions**										
Diabetes mellitus, no. (%)	N.A.	N.A.	33 (20.0)	36 (22.0)	45 (38.1)	38 (29.5)	596 (32.9)	552 (30.8)	674/2102 (32.1)	626/2088 (30.0)
Solid-organ cancer, no. (%)	64 (20.9)	58 (19.5)	0	0	32 (27.1)	32 (24.8)	400 (22.1)	382 (21.3)	496/2408 (20.6)	472/2386 (19.8)
Obesity, no. (%)	N.A.	N.A.	N.A.	N.A.	N.A.	N.A.	331 (18.2)	324 (18.1)	331/1814 (18.2)	324/1794 (18.1)
Arrhythmia, no. (%)	N.A.	N.A.	N.A.	N.A.	N.A.	N.A.	264 (14.6)	276 (15.4)	264/1814 (14.6)	276/1794 (15.4)
Chronic obstructive pulmonary disease, no. (%)	N.A.	N.A.	N.A.	N.A.	N.A.	N.A.	198 (10.9)	195 (10.9)	198/1814 (10.9)	195/1794 (10.9)
Renal insufficiency, no. (%)	N.A.	N.A.	440/503 (87.5) [d]	440/503 (87.5) [d]	18/118 (15.3)	32/129 (24.8)	217 (12.0)	208 (11.6)	455/2184 (20.8)	460/2175 (21.1)
Coronary artery disease, no. (%)	N.A.	N.A.	N.A.	N.A.	N.A.	N.A.	193 (10.6)	200 (11.1)	193/1814 (10.6)	200/1794 (11.1)
Congestive heart failure, no. (%)	N.A.	N.A.	N.A.	N.A.	N.A.	N.A.	205 (11.3)	181 (10.1)	205/1814 (11.3)	181/1794 (10.1)
Liver disease, no. (%)	N.A.	N.A.	N.A.	N.A.	10/118 (8.5)	13/129 (10.1)	117 (6.4)	110 (6.1)	127/1932 (6.6)	123/1923 (6.4)
Peripheral vascular disease, no. (%)	N.A.	N.A.	N.A.	N.A.	N.A.	N.A.	107 (5.9)	116 (6.5)	107/1814 (5.9)	116/1794 (6.5)
Dialysis dependency, no. (%)	N.A.	N.A.	N.A.	N.A.	4/118 (3.4)	8/129 (6.2)	60 (3.3)	67 (3.7)	64/1932 (33.1)	75/1923 (39.0)
Leukemia or lymphoma, no. (%)	N.A.	N.A.	0	0	N.A.	N.A.	49 (2.7)	52 (2.9)	49/1983 (2.5)	52/1959 (2.6)
Glucocorticoid use or immunosuppression, no. (%) [e]	69 (22.5)	81 (27.2)	0	0	17/118 (14.4)	14/129 (10.9)	230 (12.7)	210 (11.7)	316/2407 (13.1)	305/2386 (12.8)

[a] Scores on the Sequential Organ Failure Assessment (SOFA) range from 0 to 24, with higher scores indicating more severe organ failure.
[b] Scores on the Quick Sequential Organ Failure Assessment (qSOFA) range from 0 to 3, with higher scores indicating more severe organ failure.
[c] Charlson Comorbidity Index scores range from 0 to 37, with higher values indicating greater number or degree of underlying comorbidities.
[d] Mild, moderate, or severe renal impairment. No further details on how this distribution is divided across the study groups.
[e] Immunosuppression included chemotherapy and prednisone or equivalent glucocorticoid use of more than 15 mg per day.

Table S4 Comparative characteristics of bacteremia in the included trials

	**Yahav et al., 2019**		**von Dach et al., 2020**		**Molina et al., 2022**		**Daneman et al., 2024**		**Pooled population**	
	7-Day Group	14-Day Group	7-Day Group	14-Day Group	7-Day Group	14-Day Group	7-Day Group	14-Day Group	7-Day Group	14-Day Group
Population size, n	306	298	169	165	119	129	1814	1794	2408	2386
**SOURCE OF ACQUISITON OF BACTEREMIA**										
Community, no. (%)	225 (73.5)	203 (68.1)	108 (64.0)	99 (60.0)	49/118 (41.5)	54/129 (41.9)	1380 (76.1)	1342 (74.8)	1762/2407 (73.2)	1698/2386 (71.2)
Hospital ward, no. (%)	81 (26.5)	95 (31.9)	45 (27.0)	45 (27.0)	36/118 (30.5)	45/129 (34.9)	231 (12.7)	252 (14.0)	393/2407 (16.3)	437/2386 (18.3)
Healthcare-related	N.A.	N.A.	16 (9.0)	21 (13.0)	33/118 (28.0)	30/129 (23.3)	N.A.	N.A.	49/287 (17.1)	51/294 (17.3)
ICU, no. (%)	N.A.	N.A.	N.A.	N.A.	N.A.	N.A.	203 (11.2)	200 (11.1)	203/1814 (11.2)	200/1794 (11.1)
**SOURCE OF BACTEREMIA**										
Urinary tract, no. (%)	212 (69.3)	199 (66.8)	107 (63.0)	117 (71.0)	70/118 (59.3)	66/129 (51.2)	757 (41.7)	766 (42.7)	1146/2407 (47.6)	1148/2386 (48.1)
Intraabdominal, no. (%)	37 (12.1)	34 (11.4)	37 (22.0)	20 (12.0)	16/118 (13.6)	18/129 (14.0)	337 (18.6)	342 (19.1)	427/2407 (17.7)	414/2386 (17.3)
Lung, no. (%)	14 (4.6)	10 (3.4)	14 (8.0)	16 (10.0)	3/118 (2.5)	12/129 (9.3)	229 (12.6)	240 (13.4)	260/2407 (10.8)	278/2386 (11.7)
Vascular catheter/endovascular device, no. (%)	15 (4.9)	23 (7.7)	5 (3.0)	5 (3.0)	14/118 (11.9)	16/129 (12.4)	116 (6.4)	113 (6.3)	150/2 407 (6.2)	157/2386 (6.6)
Skin, soft tissue, or both, no. (%)	5 (1.6)	4 (1.3)	N.A.	N.A.	N.A.	N.A.	104 (5.7)	83 (4.6)	109/2120 (5.1)	87/2092 (4.2)
Other, no. (%)	N.A.	N.A.	6 (4.0)	7 (4.0)	5/118 (4.2)	6/129 (4.7)	37 (2.0)	30 (1.7)	48/2101 (2.3)	43/2088 (2.1)
Undefined or unknown, no. (%)	N.A.	N.A.	N.A.	N.A.	10/118 (8.5)	11/129 (8.5)	234 (12.9)	220 (12.3)	244/1932 (12.6)	231/1923 (12.0)
Procedures to control source of infection, no. (%)	N.A.	N.A.	N.A.	N.A.	N.A.	N.A.	795 (43.8)	826 (46.1)	795/1814 (43.8)	826/1794 (46.0)
**MOST COMMONLY ISOLATED PATHOGENS IN BLOOD CULTURES**										
Escherichia coli, no. (%)	186 (60.8)	194 (65.1)	123 (73.0)	124 (75.0)	76 (66.4)	79 (61.2)	805 (44.4)	777 (43.3)	1190/2408 (49.4)	1074/2386 (45.0)
Klebsiella species, no. (%)	47 (15.3)	33 (11.1)	35 (21.0)	26 (16.0)	23 (19.5)	23 (17.8)	273 (15.0)	279 (15.6)	378/2408 (15.7)	361/2386 (15.1)
Enterococcus species, no. (%)	N.A.	N.A.	N.A.	N.A.	N.A.	N.A.	119 (6.6)	131 (7.3)	119/1814 (6.6)	131/1794 (7.3)
Coagulase-negative staphylococci, no. (%)	N.A.	N.A.	N.A.	N.A.	N.A.	N.A.	81 (4.5)	93 (5.2)	81/1814 (4.5)	93/1794 (5.2)
Pseudomonas species, no. (%)	28 (9.2)	20 (6.7)	N.A.	N.A.	N.A.	N.A.	80 (4.4)	90 (5.0)	108/2120 (5.1)	110/2092 (5.3)
Streptococcus pneumoniae, no. (%)	N.A.	N.A.	N.A.	N.A.	N.A.	N.A.	86 (4.7)	78 (4.3)	86/1814 (4.7)	78/1794 (4.3)
Enterobacter species, no. (%)	N.A.	N.A.	3 (2.0)	1 (1.0)	11 (9.2)	15 (11.6)	80 (4.4)	77 (4.3)	84/2102 (4.0)	93/2088 (4.5)
Proteus species, no. (%)	N.A.	N.A.	7 (4.0)	6 (4.0)	N.A.	N.A.	58 (3.2)	75 (4.2)	65/1983 (3.3)	81/1959 (4.1)
Serratia species, no. (%)	N.A.	N.A.	N.A.	N.A.	3 (2.5)	4 (3.1)	38 (2.1)	48 (2.7)	41/1933 (2.1)	52/1923 (2.7)
S. pyogenes, no. (%)	N.A.	N.A.	10	N.A.	N.A.	N.A.	39 (2.1)	35 (2.0)	49/1983 (2.5)	35/1794 (2.0)
Acinetobacter species, no. (%)	2 (0.7)	4 (1.3)	N.A.	N.A.	N.A.	N.A.	24 (1.3)	16 (0.9)	26/2120 (1.2)	20/2092 (1.0)
S. agalacticae, no. (%)	N.A.	N.A.	N.A.	N.A.	N.A.	N.A.	40 (2.2)	35 (2.0)	40/1814 (2.2)	35/1794 (2.0)
Other, no. (%)	43 (14)	47 (15.8)	10 (6.0)	12 (7.0)	10 (5.0)	4 (3.1)	115 (6.3)	60 (3.3)	178/2408 (7.4)	123/2386 (5.2)
**NUMBER AND TYPE OF ORGANISMS**										
Monomicrobial, gram-negative, no. (%)	N.A.	N.A.	N.A.	N.A.	N.A.	N.A.	1299 (71.6)	1263 (70.4)	1299/1814 (71.6)	1263/1794 (70.4)
Monomicrobial, gram-positive, no. (%)	N.A.	N.A.	N.A.	N.A.	N.A.	N.A.	323 (17.8)	302 (16.8)	323/1814 (17.8)	302/1794 (16.8)
Polymicrobial	N.A.	N.A.	N.A.	N.A.	N.A.	N.A.	192 (10.6)	229 (12.8)	192/1814 (10.6)	229/1794 (12.8)
**MECHANISMS OF RESISTANCE**										
ESBL, no. (%)	N.A.	N.A.	9 (5.3)	13 (7.9)	16/118 (13.6)	12/129 (9.3)	N.A.	N.A.	25/287 (8.7)	25/294 (8.5)
AmpC, no. (%)	N.A.	N.A.	N.A.	N.A.	4/118 (3.4)	9/129 (7.1)	N.A.	N.A.	4/118 (3.4)	9/129 (7.1)
**ADDITIONAL INFORMATION**										
Any urinary device, no (%)	61 (19.9)	72 (24.2)	N.A.	N.A.	N.A.	N.A.	N.A.	N.A.	61/306 (19.9)	72/298 (24.2)
Presence of permanent urinary tract device, no (%)	42 (13.7)	58 (19.5)	3 (2.0)	5 (3.0)	15/118 (12.7)	15/129 (11.6)	N.A.	N.A.	60/593 (10.1)	78/592 (13.2)
Presence of removable urethral catheter, no (%)	19 (6.2)	14 (4.7)	7 (4.0)	24 (14.0)	N.A.	N.A.	N.A.	N.A.	26/475 (5.5)	38/463 (8.2)

Table S5 Assessment of level of certainty of evidence according to GRADE recommendations

**Outcome**		**All-cause mortality at 1 years**
Trials		4 RCTs<br>all open label
Number of patients (intention-to-treat population)		4,790<br>(2,406 vs. 2,384)
Pooled effect (95% CI)		Risk Ratio 0.93<br>95% CI 0.81 to 1.07<br>p = 0.3
Down-grading factors	Risk of bias	Some
	Imprecision	High
	Inconsistency	Low
	Indirectness	Low
	Publication bias	Low
Up-grading factors	Large magnitude of effect	No
	Dose-response gradient	N.A.
	All residual confounding would decrease magnitude of effect	N.A.
**Level of certainty**		Low

Table S6 Risk of Bias Assessment according to the Revised Cochrane risk-of-bias tool for randomized trials (RoB 2)

Study	Domain 1: Risk of bias arising from the randomization process	Domain 2: Risk of bias due to deviations from the intended interventions (effect of assignment to intervention)	Domain 2: Risk of bias due to deviations from the intended interventions (effect of adhering to intervention)	Domain 3: Risk of bias due to missing outcome data	Domain 4: Risk of bias in measurement of the outcome	Domain 5: Risk of bias in selection of the reported result	Overall risk of bias
Yahav et al. 2019	Low	Some	Some	Low	Low	Low	Some Risk
Von Dach et al. 2020	Low	Some	Some	Low	Low	Low	Some Risk
Molina et al. 2022	Low	Some	Some	Low	Low	Low	Some Risk
Daneman et al., 2024	Low	Some	Some	Low	Low	Low	Some Risk
